# Automated parametrization of small molecules within the Martini 3 coarse-grained model guided by experimental log P values

**DOI:** 10.1038/s41598-025-24757-3

**Published:** 2025-10-23

**Authors:** Maria Kelidou, Kai Steffen Stroh, Herre Jelger Risselada

**Affiliations:** 1 Physics Department, Technische Universität Dortmund, Dortmund, 44227 Germany; 2 Laboratory of Biology and Modeling of the Cell, École Normale Supérieur de Lyon, Lyon, 69007 France

**Keywords:** Biological physics, Surfaces, interfaces and thin films, Biophysical chemistry, Computational biophysics, Membrane biophysics, Single-molecule biophysics

## Abstract

Molecular dynamics simulations play an important role in investigating biological systems. However, simulating large-scale systems can be computationally expensive, which can be improved by the employment of a coarse-graining force field. This study focuses on the automated parametrization of small molecules within the CGCompiler framework. This optimization approach utilizes a mixed-variable particle swarm algorithm to avoid the manual tweaking of parameters. Particularly, the optimization focuses on matching experimentally known log P values of partitioning in water-octanol phases, reproducing atomistic density profiles in lipid bilayers, and optimizing overall shape and volume aspects of the modeled atomistic molecules. After the atomistic to coarse-grained mapping, the model’s accuracy is evaluated through a fitness function, which combines structural and dynamic targets, to accurately capture the shape and behavior of the small molecule in question. Through the investigation of the interactions between small molecules and cellular membranes, this optimization process supports the development of accurate coarse-grained models for small molecules relevant to drug discovery. Our work demonstrates promising results in automating the high-fidelity parametrization of small molecules using the Martini 3 force-field guided by experimental log P values.

## Introduction

Molecular dynamics (MD) simulations are a vital tool in the field of molecular biology and drug discovery, offering a highly-detailed insight of (bio)molecules at an atomic level^1,2^. To explore and analyze more complex systems over larger length and longer time scales, the use of coarse-grained strategies becomes essential^3^. The widespread adoption of coarse-grained force fields like the Martini model for biomolecular simulations stems from their ability to merge common chemical groups consisting of multiple heavy atoms into distinct single interaction sites^4–7^. This approach has become particularly popular because of its transferability across various applications in biomolecular science, soft matter, and nanoscience. The downside of the building block approach is that the parametrization of molecules within coarse-grained models is a highly frustrating and tedious task, as chemical groups must be encoded into one out of hundreds of predefined bead types.

Beyond ongoing efforts to create databases of already-parametrized molecules based on human parametrization efforts, such as the Martini database^6^, work is underway to fully automate this pipeline. This automation aims to enable the parametrization of existing small molecule databases widely used in pharmaceutical research for drug development purposes. To this end, multiple automated approaches have been proposed, including machine learning-based methods (e.g., graph neural networks) and artificial intelligence-driven techniques (e.g., evolutionary algorithms and swarm optimization)^8–14^. These automated approaches optimize molecular parametrization workflows, thereby accelerating drug discovery timelines through the efficient exploration of molecular configurations enabled by coarse-grained modeling methodologies^15,16^. Additionally, automated parametrization could help address the challenge of keeping up with the rapidly growing number of known compounds and targets in drug discovery. Yet, while automated approaches can generate initial parametrizations quickly, they often lack the nuanced understanding of molecular behavior that comes from careful reproduction of properties derived from atomistic simulations and experiments.

To this end, we are developing the CGCompiler^12^ approach that automizes high-fidelity (re)parametrization within the Martini 3 model using mixed-variable particle swarm optimization. This method circumvents the problem of assigning predefined nonbonded interaction types (discrete variables) while simultaneously optimizing bond length (continuous variables). By overcoming the inherent dependency between nonbonded and bonded interactions, CGCompiler performs a multiobjective optimization that matches provided targets derived from atomistic simulations as well as experimentally derived targets.

The standard parametrization procedure entails the manual setting of all initial (trial) force-field parameters and their subsequent changes to fit the desired properties. The CGCompiler requires only the initial mapping of the atomistic structure and its coarse-grained parametrization. This step can greatly benefit from the development of automated mapping schemes^13,14^, whose crude parametrization also provides a valuable starting point for refinement by CGCompiler. Afterwards a mixed-variable particle swarm optimization algorithm is employed to accomplish the molecule’s optimization, thereby overcoming the hurdles of tweaking the parameters by hand and facilitating a more accurate and efficient parametrization. The model is evaluated based on a list of properties and their target values provided by the user (fitness function).

Partition coefficients, particularly octanol-water partition coefficients, play a crucial role in small molecule and drug design^17–19^. They serve as primary indicators of hydrophobicity and membrane permeability, making them essential tools in assessing a compound’s potential as a drug candidate. Given that the octanol-water partition coefficients of common small molecules have been well experimentally determined, reproducing these coefficients represents the primary goal in guiding the parametrization of small molecules.

In addition to partition coefficients, atomistic density profiles within lipid bilayers provide a complementary and membrane-specific target for parametrization. Unlike bulk partitioning, density profiles investigate the spatial distribution and orientation of molecules across the heterogeneous lateral membrane interface directly, capturing interactions with different chemical groups within the lipid and the insertion depth within the bilayer^20–23^. Incorporating such information allows coarse-grained models to more precisely account for additional structural and electrostatic effects that are often absent when optimizing solely against octanol–water partitioning free energies. Furthermore, the density profiles of individual beads correspond to the orientation of molecules in the membrane, enabling more precise parametrization of the local molecular chemistry within molecules that are not uniquely determined by log P values alone.

For this purpose, we extended CGCompiler to optimize molecules based on the free energy of transfer between octanol and water phases, as well as based on the atomistic density profiles within lipid bilayers. We also incorporated a scheme for the bonded parameters to simultaneously match the Solvent Accessible Surface Area (SASA). Our focus is on the parametrization of dopamine and serotonin, two biologically highly relevant neurotransmitters. Their roles in mediating both physiological and psychological processes make them important targets for parametrization^24–27^. Furthermore, the investigation of the interactions between dopamine and serotonin and cellular membranes, as well as their receptors, is fundamental to understanding and treating a variety of neurological disorders^28,29^.

We report a significant advance in the automated parametrization of small molecules within the Martini 3 force field by extending CGCompiler to simultaneously optimize against experimental log P values and atomistic density profiles in lipid bilayers. The inclusion of the density profiles of mapped interaction sites provides a direct membrane-specific target alongside bulk partitioning data, ensuring more accurate reproduction of molecular orientation and insertion behavior at biologically relevant interfaces. Incorporating diverse targets improves the accuracy of membrane interaction modeling and enhances the capability of coarse-grained parametrization to account for subtle but biologically relevant effects, such as electrostatic interactions(the presence of net charge) and local molecular chemistry.

## Methods

**Fig. 1:**
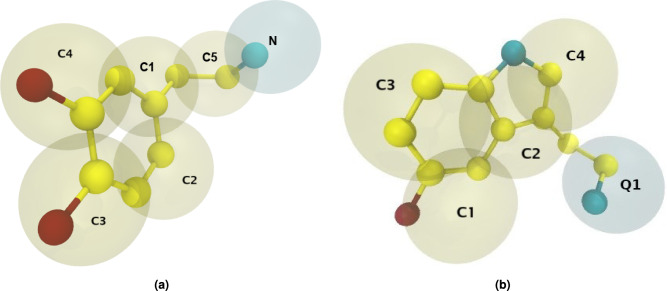
Snapshot of the coarse-grained models of dopamine (**a**) and serotonin (**b**). Atoms are colored by element type, while the blue coarse-grained bead marks the charged group. The bead labels serve as references to subsequent figures.

The initial step in the coarse-graining process involved determining the grouping of atoms into beads. For dopamine, the process was carried out by hand and the result can be seen in Fig. 1. For the initial mapping of serotonin, we used Auto-Martini^13^, but a finer adjustment of the parameters was necessary for Martini 3.

### CGCompiler

**Fig. 2:**
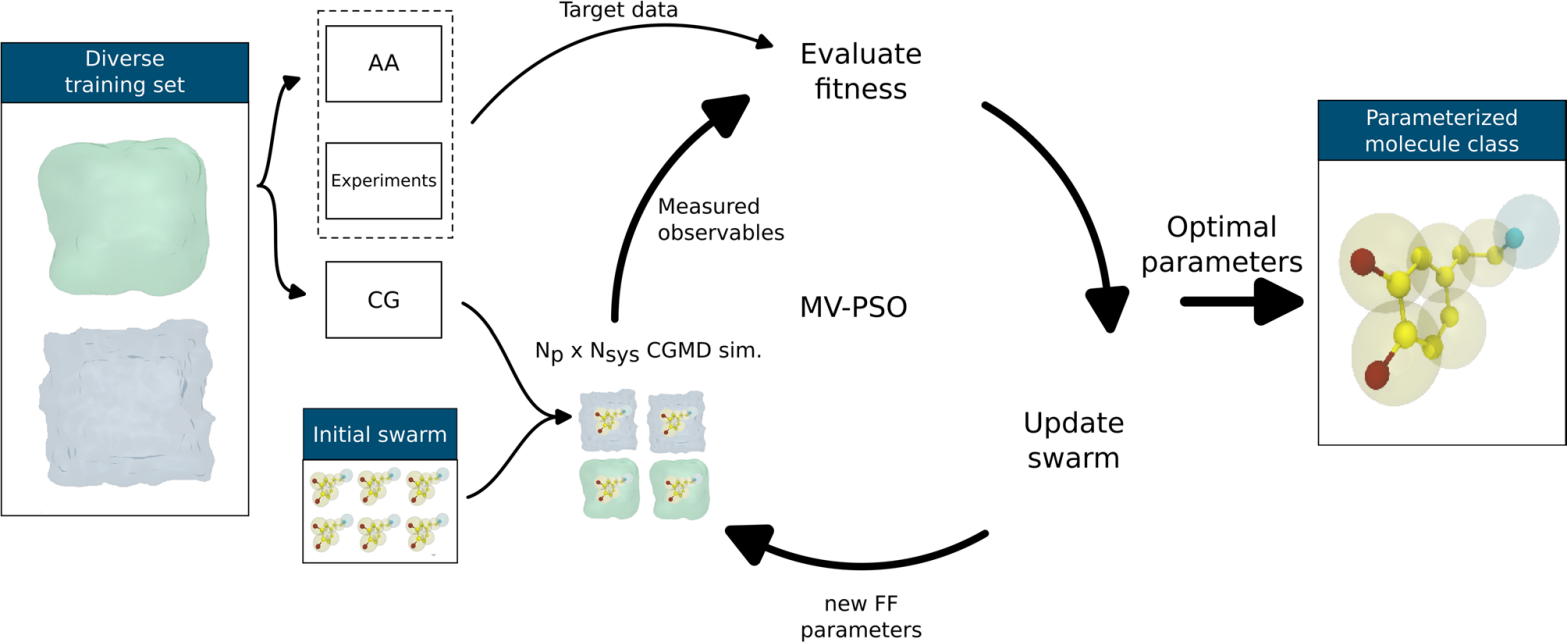
Representation of the CGCompiler framework, adapted from^12^. The octanol training system is portrayed by a mint green colour, while water is portrayed by a light blue colour.

Small molecule parametrization in Martini 3 requires careful adjustment of many parameters to match several goals. Identifying the right parameters to improve specific behaviors, especially in complex interactions, is a difficult and time-consuming task. Automation becomes crucial for handling large molecule databases, organizing the parametrization process into a clear, hierarchical system. One automated method is Particle Swarm Optimization (PSO), known for efficiently finding the best solutions in complex, multidimensional spaces. PSO is ideal for optimizing continuous variables in coarse-grained models, though it faces challenges with predefined, discrete parameters in building block models like Martini. The CGCompiler Python package^12^ provides efficient coarse-grained molecule parametrization through mixed-variable particle swarm optimization. This method optimizes both categorical (predefined bead types) and continuous (bonds, angles, dihedrals, etc.) variables simultaneously. Built on the GROMACS simulation engine^30–33^, CGCompiler substantially simplifies force field parametrization, particularly for building-block approaches.

The parametrization workflow, which can be seen in Fig. 2, involves selecting mapping and bead sizes, assigning chemical bead types, and choosing bonded terms and parameters. The presented algorithm optimizes bead size, chemical bead type, and bonded parameters simultaneously. The workflow includes the user providing the target data and creating a set of CG training systems. The optimization algorithm iteratively generates candidate solutions, runs MD simulations, scores solutions based on how well targets are reproduced, updates solutions using the swarm’s knowledge, and repeats until termination criteria are met.

The parametrization of small molecules made it imperative to introduce new targets and devise alternative methodologies for their calculation within the domain of the CGCompiler. As small molecules are much smaller and more flexible than proteins or lipids, additional metrics are needed to capture their physical properties. One of the relevant targets for small molecules is the Solvent Accessible Surface Area (SASA). It is a widely used metric in molecular biology and computational chemistry that quantifies the extent of a molecule’s surface that is accessible to a solvent^34^. This measurement is crucial in understanding the interactions, dynamics, as well as the structures of biomolecules in various environments. The SASA target value was obtained through the GROMACS tool gmx sasa, computed as an average through high-sampling atomistic simulations of each small molecule. Due to the reduced resolution of coarse-grained models, perfect agreement with atomistic SASA is not expected. Nonetheless, including SASA as an objective provides a useful guide for capturing the overall molecular shape and solvent-exposed surface during parametrization.

Investigating the behavior and thermodynamic properties of small molecules across different solvents is an important task, which is often expressed through the partition coefficient or equivalently log P value. Therefore, we implemented the calculation of the partition coefficient into the CGCompiler through the application of the Multistate Bennett Acceptance Ratio (MBAR) method^35,36^. This approach allowed us to accurately compute the necessary free energy of transfer for determining the partition coefficient, as defined by the equation adapted from^37^ to account for the free energy transfer from octanol to water instead of water to octanol:

1 $$\begin{aligned} \text {log}P=\frac{\Delta G_{\text {transfer}}}{RT\text {ln}(10)} \end{aligned}$$

where

2 $$\begin{aligned} \Delta G_{\text {transfer}}=\Delta G_{o\rightarrow g} - \Delta G_{w \rightarrow g} \end{aligned}$$

The calculation of solvation free energies in octanol and water typically employs a thermodynamic cycle involving transfer to the gas phase to establish a system-independent reference state. However, this approach presents significant challenges in accuracy. The transfer free energy ($\Delta G_{\text {transfer}}$) is computed as the difference between $\Delta G_{o\rightarrow g}$ and $\Delta G_{w \rightarrow g}$, where the subscripts $o \rightarrow g$ and $w\rightarrow g$ denote transfer to the gas phase. These individual terms involve large values of several hundred kJ/mol due to the switching off of non-bonded interactions during the alchemical transformation. As a result, typical sampling errors of several kJ/mol become comparable to the magnitude of $\Delta G_{\text {transfer}}$ itself, rendering the calculations inherently inaccurate and computationally expensive for high-throughput applications.

To circumvent these limitations, we implement a chemical perturbation scheme utilizing a fixed reference topology with a predetermined $\Delta G_{o\rightarrow w}^{\text {ref}}$ value^38^. This approach enables the calculation of transfer free energies for newly parametrized molecules according to the equation:

3 $$\begin{aligned} \Delta G_{o \rightarrow w}^{\text {new}} = \left( \Delta G_{o\rightarrow g}^{\text {ref}} - \Delta G_{w \rightarrow g}^{\text {ref}}\right) + \left( \Delta \Delta G_{w}^{\text {ref} \rightarrow \text {new}} - \Delta \Delta G_{o}^{\text {ref} \rightarrow \text {new}} \right) \end{aligned}$$

where $\Delta \Delta G^{\text {ref} \rightarrow \text {new}}$ represents the relative free energy difference between new molecule parameters and reference parameters, obtained through on-the-fly chemical perturbation, and $\Delta G^{\text {ref}}$ denotes the known reference free energies of the predefined reference molecule. It is however important to emphasize that precise determination of $\Delta G^{\text {ref}}$ is crucial as it establishes the fundamental reference point for all subsequent log P estimations obtained via chemical perturbation and therefore largely determines the systematic error.

Optimizing parametrization solely on the octanol-water partitioning free energies may lack several key membrane-specific interactions, including electrostatic effects with lipid headgroups and the ordered structural characteristics of the membrane interface. Therefore, we investigated the interfacial behavior of small molecules at lipid membranes by calculating local density profiles within a coarse-grained POPC membrane, as an additional objective in the multi-objective optimization scheme. These calculations were compared against atomistic simulations of our small molecules using the CHARMM36 force field, computed through the GROMACS tool gmx density. It is important to symmetrize the density profiles, as the slow binding and unbinding kinetics of small molecules can result in highly asymmetric profiles, skewing the density matching process. As the leaflet affinity is by definition identical for a symmetric bilayer, symmetrizing the density profile is fully justified.

The convergence behavior in swarm optimization algorithms exhibits a direct correlation with problem dimensionality. As the number of dimensions increases, maintaining sufficient population diversity requires proportionally larger swarm sizes. In our implementation, we employed swarm sizes of 72 particles for dopamine-related parameters (six interaction sites) and 48 particles for serotonin-related parameters (five interaction sites), balancing computational efficiency with the molecular complexity of each system. These specific choices of parameters were guided by a balance between computational efficiency and convergence quality. Larger swarms tend to improve global search capabilities, but beyond certain sizes, the computational cost is not reflected in the convergence quality. We implemented a consistent optimization protocol across both systems, utilizing 50 iterations per convergence cycle. Equal weights (1.0) were assigned to objective functions within each system to maintain balanced optimization dynamics.

### Fitness function

The CGCompiler evaluates parametrization performance using a cost function.

4 $$\begin{aligned} \text {cost} = \sum _{o} w_o f_o \end{aligned}$$

This function aims to be minimized and consists of multiple normalized objective functions ($f_o$), each assigned a user-defined weight ($w_o$). These weights enable users to prioritize and balance the significance of various parametrization goals. In our parametrization procedure we used four objective functions of equal weights, namely SASA, bond distributions^39,40^, the octanol-water partition coefficient, and mapped-bead density distributions.

### Atomistic simulations

For the generation of target bond and density distribution data, we conducted atomistic simulations of the small molecule in a 3nm cubic box of water ($\approx$ 900 molecules) and in a (6, 6, 8)nm box of water-POPC ($\approx$ 6400 water and 85 POPC molecules) using the CHARMM36 force field^41–43^. After an energy minimization and NPT equilibration of 100ns, followed a production run of 1$\mu$s. During production, we used a time step of 2fs, the velocity-rescale algorithm as the thermostat with a coupling time of $\tau _t=0.1$ps, and the Parinnelo-Rahman as a barostat with a coupling time of $\tau _p=2.0$ps.

### Coarse-grained simulations

In the CGCompiler, we conducted three parallel sets of simulations of the small molecules in GROMACS 2023.2; once in water, once in two separate boxes of water and octanol for the free energy calculations, and once in water-POPC membrane. The same models of small molecules were used and evaluated in all training systems. In the first system, we chose the fitness as a function of SASA and the bond distribution data. In the second system, we used the value of log P as the fitness and we compared it to the value from experimental data listed in Table 1. In the third training system, we chose the fitness as a function of the mapped-bead density distribution. In the first and third training systems, we obtained the reference data from our high-sampling atomistic simulations.

For the first and third training system, we conducted the required energy minimization and two stages of NPT equilibration with time steps $dt=2$fs and 20fs, followed by a production run of 400ns with a time step of 20fs. During production, we used the velocity-rescale algorithm as the thermostat with a coupling time of $\tau _t=1$ps, and the Parinnelo-Rahman as a barostat with a coupling time of $\tau _p=12.0$ps. For the subsequent free energy calculation simulations, we chose the following lambda states: 0.0, 0.1, 0.2, 0.3, 0.4, 0.5, 0.55, 0.6, 0.63, 0.65, 0.68, 0.7, 0.72, 0.73, 0.75, 0.77, 0.8, 0.85, 0.9, 0.95, 1.0. These simulations entailed a production run of 10ns for each different lambda state, using the same settings as in the initial training system.

## Results

**Fig. 3:**
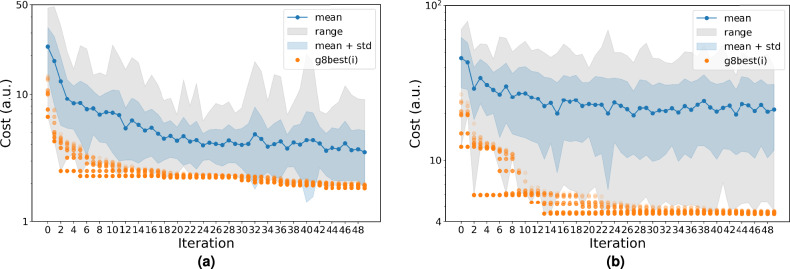
Cost function convergence for CG dopamine (**a**) and serotonin (**b**). g8best indicates the 8 best candidate solutions of the swarm. The mean value of the cost function of the whole swarm is portrayed in blue scatter points, while the entire range of cost function values is shown as a shaded gray region.

The CGCompiler generates optimized dopamine and serotonin parameters in .itp format. Figure 3 shows the convergence behavior of CGCompiler’s total cost function across 50 iterations. This composite metric combines four normalized objective functions: bond distributions, solvent accessible surface area (SASA), octanol-water partition coefficient ($\log P_{OW}$), and density distributions.

**Fig. 4:**
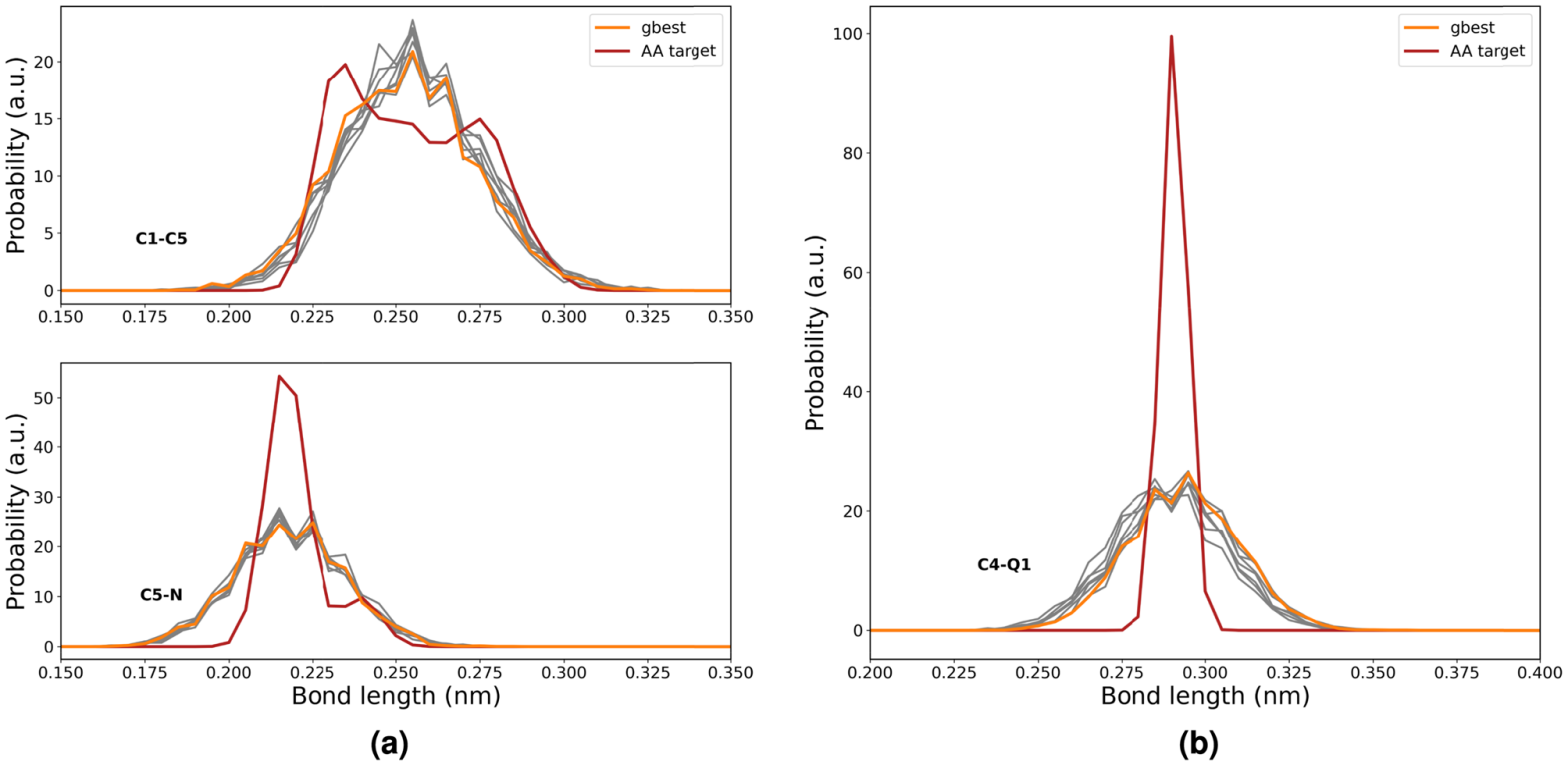
Bond distribution comparison between AA target data and CGCompiler output of the beads in the dopamine **(a)** and serotonin tail **(b)**. The bonds are between the beads that are labeled in Fig. 1. gbest portrays the best candidate solution. The next best candidate solutions are portrayed in gray.

In Fig. 4, we have plotted the bond distribution comparison between the best candidate solutions from the CGCompiler with the atomistic reference data. Distribution overlap was optimized using the earth mover’s distance criterion^12^. The Earth Mover’s Distance (EMD) is a measure of dissimilarity between two probability distributions that captures the minimum amount of work needed to transform one distribution into another. Using EMD rather than peak fitting has the advantage of effectively capturing both the peak position and width of a distribution within a single parameter. In our dopamine model the bonds are between beads C1-C5 and C5-N, which are located in the dopamine tail. In our serotonin model, the bond is between C4-Q1, where Q1 is the serotonin tail. The relevant beads are labeled in Fig. 1. We can see that there is good agreement with the means of the atomistic target distributions though the width of the distribution generally tends to be somewhat wider within the coarse-grained model particularly in case of C4-Q1. Though a stronger force constant would result in a narrower bond distribution, it may potentially compromise simultaneous optimization of other objectives, including SASA calculations that determine molecular shape and volume and even log P values and local density profiles. We, however, note that the optimization outcome represents the best balance among four objectives rather than optimal performance for any single objective.

**Table 1:** Comparison of experimental and computational partition coefficient value. Experimental values were taken from^44,45^. The errors represent the standard deviation of the free energy difference, propagated to log P.

log*P*	Dopamine	Serotonin
Experimental Value	-0.99	0.21
CGCompiler Value	$-1.008 \pm 0.004$	$0.211 \pm 0.002$

**Fig. 5:**
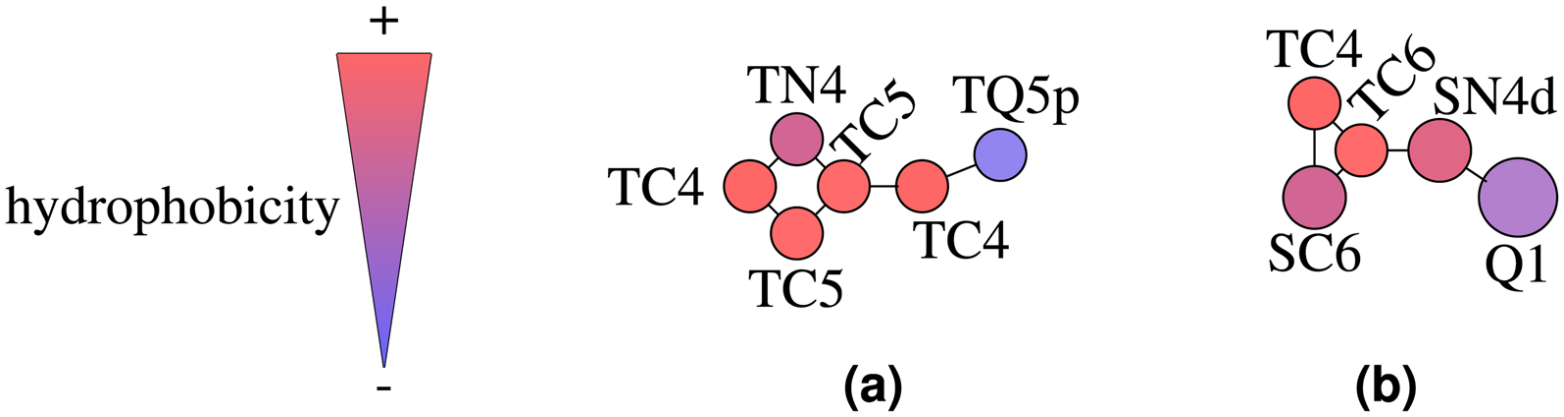
Visualization of the hydrophobicity scale for bead types in the optimized CG models of dopamine **(a)** and serotonin **(b)**, adapted from^46^.

The log P values presented in Table 1 demonstrate strong agreement between experimental and calculated values, indicating that the models are expected to effectively capture the overall oil-water partitioning tendencies of these small molecules, including their insertion behavior within biological lipid membranes. The composition of the optimized molecules and their relative hydrophobicity can be seen in Fig. 5.

**Fig. 6:**
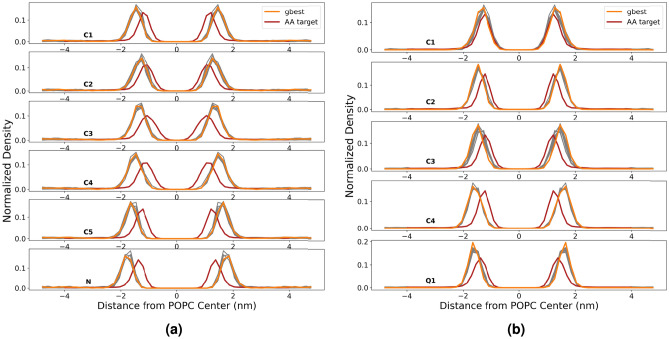
Direct density comparison of mapped beads in atomistic and coarse-grained dopamine (**a**) and serotonin (**b**) as a function of the distance from the center of the POPC membrane. gbest portrays the best candidate solution. The next best candidate solutions are portrayed in gray.

In biology, dopamine and serotonin tend to bind strongly to lipid membranes^47,48^. In our simulations, as can be seen in Fig. 6, the density profile of dopamine shows peak positions at slightly shallower insertion depths compared to the atomistic reference. Serotonin exhibits a closer matching of insertion depths overall, although there is still a noteworthy shift toward shallower insertion depths. Both molecules show slightly elevated binding energies compared to the atomistic reference.

**Fig. 7:**
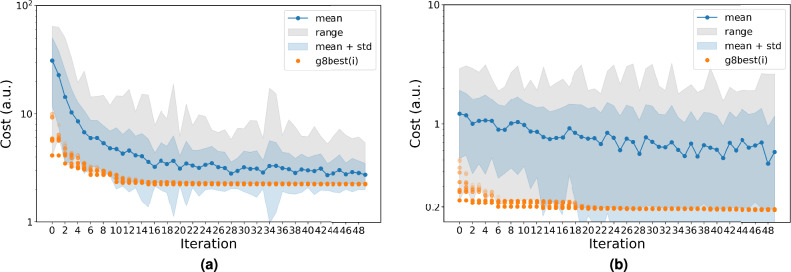
Cost function convergence for CG pyrrolidine (**a**) and phenol (**b**). g8best indicates the 8 best candidate solutions of the swarm. The mean value of the cost function of the whole swarm is portrayed in blue scatter points, while the entire range of cost function values is shown as a shaded gray region.

To validate our automated parametrization approach against human efforts, we parametrized small molecules that are already available in the Martini 3 database^6^. For dopamine and serotonin there still exists no corresponding human-made model. Pyrrolidine and phenol were selected as small molecules because they are small, favorably interact with lipid membranes, and somewhat resemble dopamine and serotonin. As a starting point of the optimization we used the corresponding models from the Martini 3 small molecule database. We followed the same parametrization protocol as with dopamine and serotonin, maintaining the same components of the cost function, with the exception of phenol, whose ring structure is based on bond constraints of a fixed length (no bond distribution). Target partition coefficients for both molecules were obtained from the PubChem XLogP3 3.0 tool^49^. Figure 7 shows the convergence behavior of CGCompiler’s total cost function across 50 iterations.

**Table 2:** Comparison of predicted, CGCompiler, and Martini 3 database partition coefficient value. Predicted data were taken from^49^. The errors represent the standard deviation of the free energy difference, propagated to log P.

log*P*	Pyrrolidine	Phenol
Predicted Value	0.5	1.5
CGCompiler Value	$0.5486 \pm 0.0015$	$1.4786 \pm 0.0018$
M3 Database Value	$0.859 \pm 0.0015$	$0.590 \pm 0.0017$

**Fig. 8:**
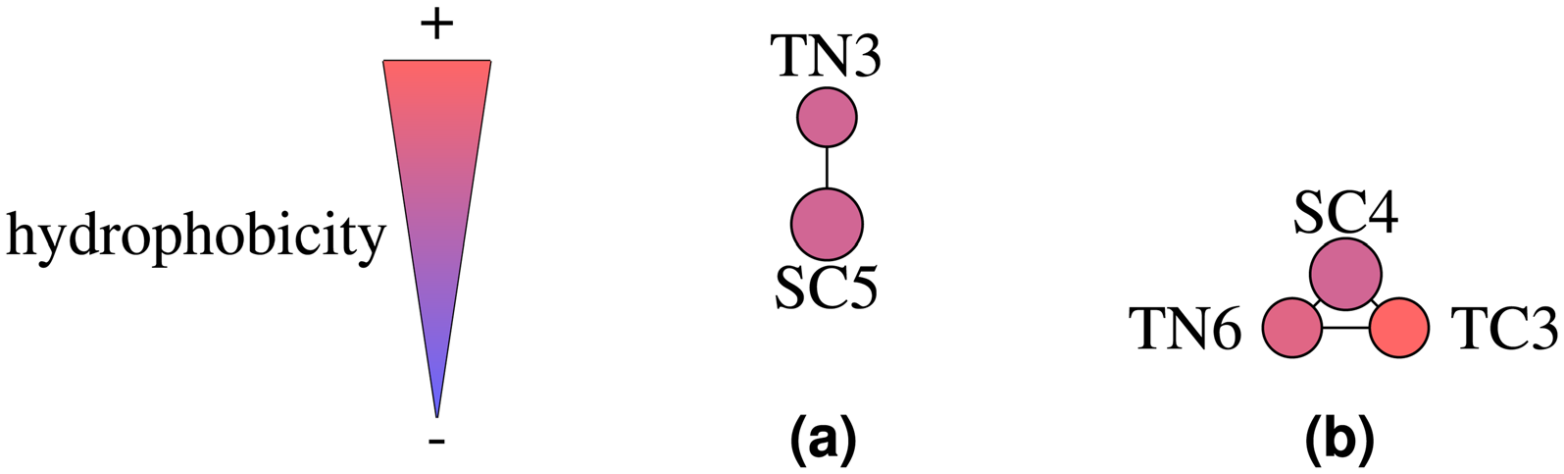
Visualization of the hydrophobicity scale for bead types in the optimized CG models of pyrrolidine **(a)** and phenol **(b)**, adapted from^46^.

The CGCompiler log P values presented in Table 2 are sufficiently close to the predicted values, indicating that the models are expected to effectively capture the overall oil-water partitioning tendencies of pyrrolidine and phenol. The composition of the optimized molecules and their relative hydrophobicity can be seen in Fig. 8.

**Fig. 9:**
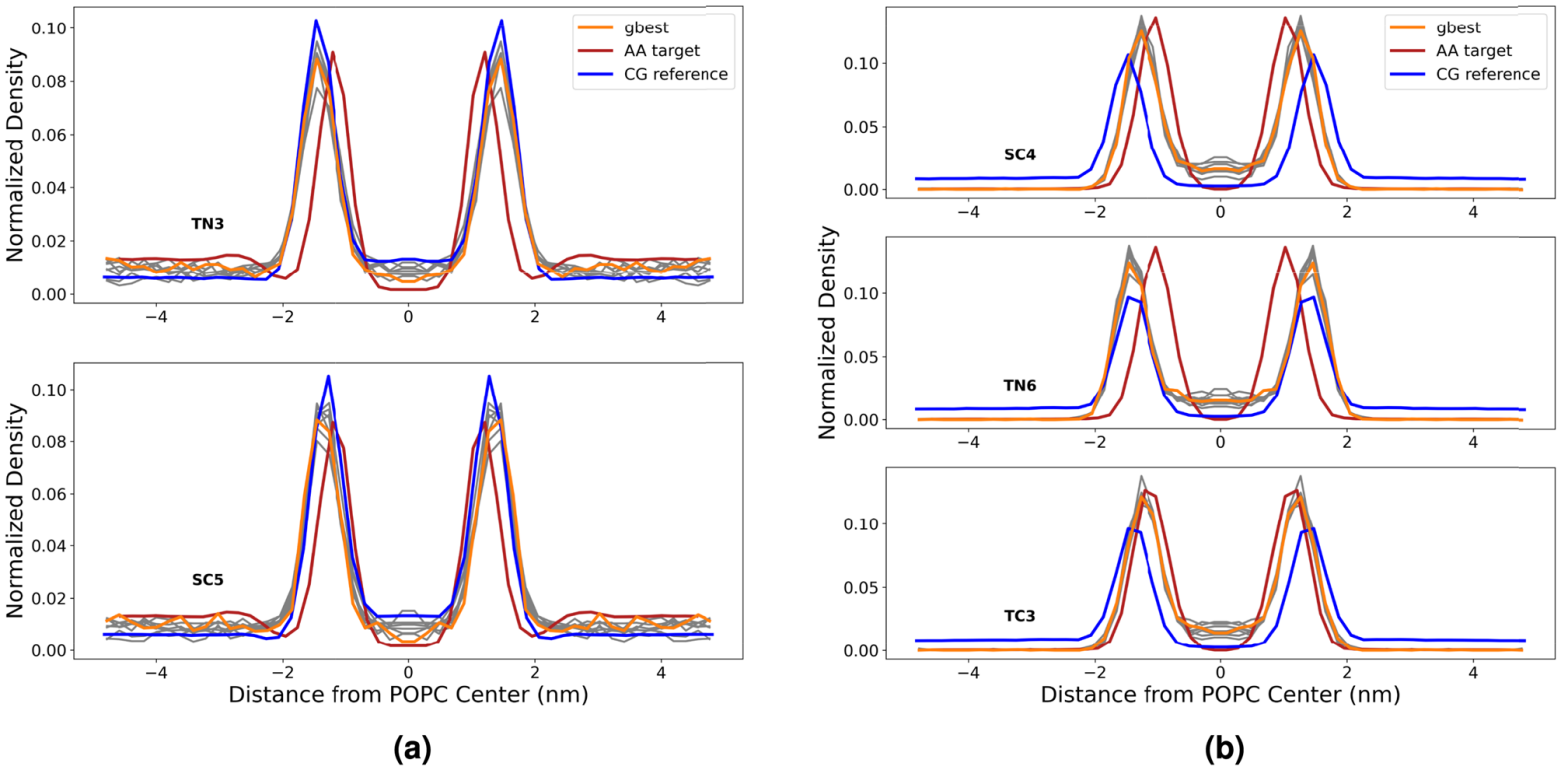
Direct density comparison of mapped beads in atomistic, CGCompiler output and coarse-grained Martini 3 database pyrrolidine (**a**) and phenol (**b**) as a function of the distance from the center of the POPC membrane. The next best candidate solutions are portrayed in gray.

In Fig. 9, pyrrolidine membrane insertions match well with the atomistic reference, although the human-made coarse-grained reference (CG reference) exhibits elevated values. However, the insertion depth of bead SC5 shows slightly poorer agreement with the human-made coarse-grained reference based on peak position. Based on peak height and concomitant distribution width, however, performance is better. This is because the EMD criterion considers all overall distribution features, not just the peak position. For phenol, both insertion depths and binding energies display closer agreement overall. The human-made phenol model is clearly too hydrophilic, as evidenced by a log P value that is too small and membrane insertion that is too shallow.

For both molecules, a consistent tendency toward shallower insertion depths compared to atomistic simulation remains, similar to what was previously observed for dopamine and serotonin. This suggests that matching log P values natively results in a somewhat shallower membrane insertion and therefore molecules behave effectively too hydrophilic when interacting with lipid membranes. Interestingly, this tendency aligns with recent reports of overly hydrophilic protein-membrane interactions in Martini 3^50–52^, indicating that this issue may extend beyond amino acids. Some care must be taken, as our log P value simulations were based on dry octanol, in accordance with Ref.^37^, whereas the human-based model used hydrated octanol containing a 0.3 mole fraction of water^6^. For small molecules with log P values close to 0 (e.g. pyrrolidine with a log P value of 0.5), the difference in solvation free energy between wet and dry octanol is expected to be negligible.

**Fig. 10:**
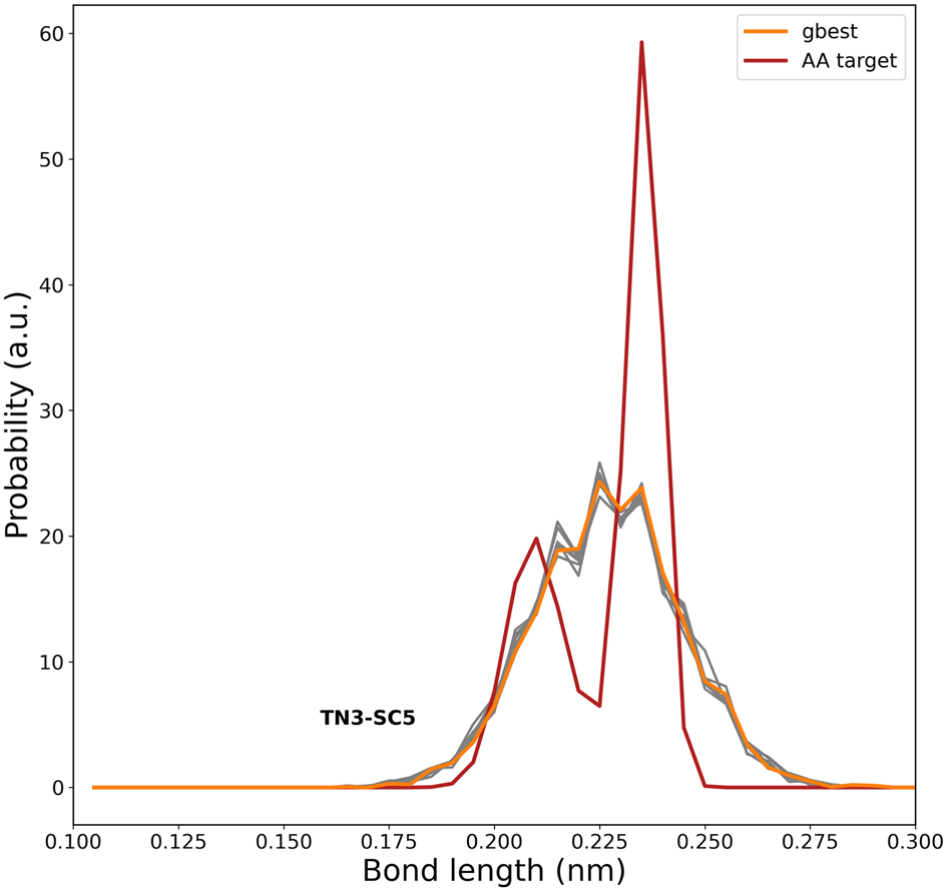
Bond distribution comparison between AA target data and CGCompiler output of the beads in pyrrolidine. gbest portrays the best candidate solution. The next best candidate solutions are portrayed in gray.

Finally, in Fig. 10, we plot the bond distribution comparison between the best candidate solutions from CGCompiler and the atomistic reference data. The overlap of the distributions was optimized using the Earth Mover’s Distance criterion^12^. As can be seen, there is good agreement with the mean of the atomistic target distribution.

## Discussion

The parametrization of molecules within building block coarse-grained models is a highly laborious and tedious task, as chemical groups must be encoded into one out of hundreds of predefined bead types. Recent advances in computational chemistry have led to the development of several automated approaches for molecular parametrization, including machine learning-based methods and artificial intelligence-driven techniques^8–14^. Building upon our CGCompiler framework^12^, we have enhanced the parametrization capabilities within the Martini 3 force field through the integration of mixed-variable particle optimization. This advancement specifically targets high-fidelity parametrization of small molecules by incorporating experimental partitioning data, atomistic density profiles, and molecular volume/shape considerations into the optimization process. The current implementation demonstrates strong potential for automated parametrization of small molecules in the Martini 3 force field, offering significant advantages over ongoing manual parametrization efforts^6^. It enables precise molecular characterization through systematic integration of experimental partitioning data with structural and dynamical information from atomistic simulations regarding molecular flexibility, volume, and shape. This simultaneous optimization of multiple competing objectives can exceed human capabilities.

The octanol-water partition coefficient represents a fundamental metric in molecular characterization, providing essential insights into solubility properties across different solvents and interfacial behaviors. As a cornerstone of building-block coarse-grained force field methodology, the log P value delivers a comprehensive measure of molecular partitioning. While this metric offers valuable predictions regarding membrane permeation and insertion properties, solely parametrizing molecules based on reproducing log P values faces two critical limitations: (i) Chemical locality: The log P value contains limited information about chemical locality effects across the molecule, particularly concerning hydrophobicity distribution around interaction sites. (ii) Effect of charge: Charged molecules such as serotonin and dopamine exhibit amphiphilic nature at the octanol-water interface, yet the explicit effect of charge itself is not captured in the parametrization due to the absence of partial charges within the coarse-grained model.

To address these limitations while maintaining accurate log P values, we have additionally implemented local density profile comparison of mapped beads within lipid membranes as an additional objective function in CGCompiler. The lipid membrane interface provides a more physiologically relevant environment and features additional interactions with zwitterionic head groups as well as the presence of a distinct liquid crystalline ordering. Although experimental measurements of membrane-molecule interactions remain more challenging to obtain than octanol-water partitioning data, this limitation can be effectively bridged through strategic application of atomistic simulations. In our optimization framework, experimental octanol–water partitioning free energies are reproduced alongside atomistic density profiles of membrane interactions, ensuring accurate parametrization of both bulk partitioning and membrane-specific behaviors. This dual-target strategy enhances predictive accuracy while maintaining computational tractability by leveraging the complementary strengths of experimental and atomistic references.

Our computational analysis shows that combining log P values with accurately reproducing local density profiles for individually mapped beads in coarse-grained simulations provides valuable insight into the overall molecular orientation and behavior at membrane interfaces. This serves as a benchmark for model quality. However, the question remains as to which matched features are most important for the quality of the model, as well as how to define model quality. For now, this is still human-determined. In our current simulations, we assigned the same weight to matching bond distributions, log P values and membrane density profiles. We observed that matching (dry) octanol log P values results in a tendency for shallower membrane insertion than in atomistic simulations. This is consistent with an inherent more hydrophilic nature. Similarly, precise matching of density profiles is anticipated to result in molecules that are inherently too hydrophobic, according to their log P value in (dry) octanol. Our log P value simulations were based on dry octanol according to the puristic physical chemical standard, other studies often include a 0.3 mole fraction of water conform with more common pharmaceutical practices. However, when both are available, we would argue that dry octanol log P values should always be preferred to wet octanol log P values. This is because coarse-grained models are unable to model either the local interfacial structure or the substantial concomitant entropic surfactant effects caused by water-octanol micellization, which significantly affects the solvation of small molecules^53^.

Ultimately, due to the inherent uncertainty surrounding the accuracy of the modeled reference systems, it is surprisingly difficult to make a fair comparison of model quality. Within our limited framework of reference, the resulting optimized models performed better overall than human-made models, which is not surprising given that optimization aims to improve performance within such a framework. In this study, equal weights (1.0) were initially assigned to all objective functions. Thoroughly optimizing these weights would require a computationally demanding process that is beyond the scope of this study. As part of the force field’s philosophy, matching some targets, such as log P values, may be deemed more essential than matching others, such as bond distributions, whose width tends to deviate inherently from atomistic simulations. This choice of weighting could be improved in future studies to better align with the philosophy of force fields^54^. However, the quality of the (automated) parametrization remains natively restricted by a limited, human-defined target set. The models that provide the best fit within that benchmarking subset are not necessarily the models that perform best in other domains. This is the prevailing problem in force-field parametrization. It is debatable whether a model optimized for most of the domains can be considered optimal when it performs more weakly in an individual domain of interest. Similarly, we anticipate that our models will natively perform best in the area of lipid membrane interactions with small molecules, as well as the subsequent change in membrane properties^55^.

While the automated high-fidelity parametrization of small molecules using mixed-variable swarm optimization represents a significant technological advance, it remains a computationally intensive endeavor that requires substantial computational resources. Even with access to dedicated computing infrastructure, parametrization of individual molecules necessitates several days of computational time. Consequently, systematic application of this methodology to extensive molecular databases containing millions of compounds is computationally prohibitive. Instead, we envision its primary utility in research contexts requiring highly accurate coarse-grained models for focused studies involving smaller sets of specifically targeted small molecules.

## Supplementary Information

Supplementary Information.

## Data Availability

The itp files for dopamine, serotonin, pyrrolidine and phenol are provided in the appendix. Remaining datasets generated during and/or analyzed during the current study are available from the corresponding author on reasonable request.
