# Supplementary material for: Automated parametrization of small molecules within the Martini 3 coarse-grained model guided by experimental log P values
Source: Sci Rep. 2025 Oct 23;15:37169. doi: 10.1038/s41598-025-24757-3 (PMC12550069; doi:10.1038/s41598-025-24757-3)
Supplement: Supplementary file 1 — Supplementary Information. [file 41598_2025_24757_MOESM1_ESM.pdf]

Appendix: GROMACS .itp Parameters for Dopamine, Serotonin, Pyrrolidine, Phenol

Dopamine

```
[ moleculetype ]
; molname    nrexcl
DOPA       1

[ atoms ]
; id     type   resnr  resname atomname cgnr charge
   1      TC5      0   DOPA     C1      1   0.00000
   2      TC5      0   DOPA     C2      2   0.00000
   3      TC4      0   DOPA     C3      3   0.00000
   4      TN4      0   DOPA     C4      4   0.00000
   5      TC4      0   DOPA     C5      5   0.00000
   6     TQ5p      0   DOPA      N      6   1.00000

[ bonds ]
;   i    j    funct   r0   fc
    1     5   1   0.24186   5039
    5     6   1   0.21231   9000

[ constraints ]
;   i    j    funct   r0
    1     2   1   0.30987
    1     3   1   0.30566
    1     4   1   0.29030
    2     3   1   0.31500
    3     4   1   0.28353

[ dihedrals ]
; ai aj ak al funct phi k  mult
    4     1     3 2   2   180.000    100

[ exclusions ]
; ai aj ak
    2     4
```

Serotonin

```
[ moleculetype ]
; molname    nrexcl
SERO       2

[ atoms ]
; id     type   resnr  resname atomname cgnr charge
   1      SC6      1   SERO     C1      1   0.00000
   2      TC4      1   SERO     C2      2   0.00000
   3      TC6      1   SERO     C3      3   0.00000
   4     SN4d      1   SERO     C4      4   0.00000
   5       Q1      1   SERO     Q1      5   1.00000

[ bonds ]
;   i    j    funct   r0   fc
    4     5   1   0.29484  1e+04

[ constraints ]
;   i    j    funct   r0
    1     2   1   0.29907
    1     3   1   0.32272
```

```
    2     3   1   0.25814
    2     4   1   0.26664
    3     4   1   0.41649

[ angles ]
; i   j   k   funct   theta0  fc
    2     4     5   2    61.820   44.6

[ dihedrals ]
; ai aj ak al funct phi k  mult
    1     3     2 4   2   180.000     10
```

Pyrrolidine

```
[ moleculetype ]
; molname    nrexcl
PYLI      1

[ atoms ]
; id     type   resnr  resname atomname cgnr charge
   1      TN3      1   PYLI     N1      1   0.00000
   2      SC5      1   PYLI     R2      2   0.00000

[ bonds ]
;   i    j    funct   r0   fc
    1     2   1   0.22677   9000
```

Phenol

```
[ moleculetype ]
; molname    nrexcl
PHEN      1

[ atoms ]
; id     type   resnr  resname atomname cgnr charge
   1      SC4      1   PHEN     O1      1   0.00000
   2      TN6      1   PHEN     R2      2   0.00000
   3      TC3      1   PHEN     R3      3   0.00000

[ constraints ]
;   i    j    funct   r0
    1     2   1   0.35000
    2     3   1   0.35000
    1     3   1   0.35000
```
